# Investigating the Magnitude and Persistence of COVID-19–Related Impacts on Affect and GPS-Derived Daily Mobility Patterns in Adolescence and Emerging Adulthood: Insights From a Smartphone-Based Intensive Longitudinal Study of Colorado-Based Youths From June 2016 to April 2022

**DOI:** 10.2196/64965

**Published:** 2025-03-17

**Authors:** Jordan D Alexander, Kelly A Duffy, Samantha M Freis, Sy-Miin Chow, Naomi P Friedman, Scott I Vrieze

**Affiliations:** 1 Department of Psychology University of Minnesota Minneapolis, MN United States; 2 Institute for Behavioral Genetics University of Colorado Boulder Boulder, CO United States; 3 Department of Psychology and Neuroscience University of Colorado Boulder Boulder, CO United States; 4 College of Health and Human Development The Pennsylvania State University University Park, PA United States

**Keywords:** adolescence, emerging adulthood, intensive longitudinal assessment, COVID-19, affect, GPS, mobility patterns, smartphone data, respiratory, infectious, pulmonary, pandemic, adolescents, teens, teenagers, mobility, apps, smartphones, intensive longitudinal panel studies, emotional well-being, well-being, daily routines, affect survey

## Abstract

**Background:**

The onset of the COVID-19 pandemic in early 2020 introduced unprecedented disruptions impacting the emotional well-being and daily routines of US youths. However, the patterns and persistence of these impacts over the pandemic’s multiyear course remain less well understood.

**Objective:**

This study examined longitudinal changes in affect and daily mobility patterns observed in adolescence and young adulthood from June 2016 to April 2022. The study aimed to quantify changes in youths’ mood and daily routines following the pandemic’s onset and in response to local COVID-19 case rates as well as the persistence of these effects over the pandemic’s multiyear course.

**Methods:**

Colorado-based adolescent and young adult twins (N=887; n=479, 54% female; mean_age_ 19.2, SD_age_ 1.5 years on January 01, 2020) participating in the CoTwins study between June 2016 and April 2022 were followed via a smartphone app, which recorded persistent GPS location data and, beginning in February 2019, administered an abbreviated Positive and Negative Affect Schedule every 2 weeks. Nonlinear trajectories in affect and daily mobility over time and in response to local COVID-19 counts were modeled via generalized additive mixed models, while the magnitude and persistence of pandemic-related changes were quantified via linear mixed effects regressions.

**Results:**

Between January and April 2020, participants experienced a 28.6% decline in daily locations visited (from 3.5 to 2.5; SD 0.9) and a 60% reduction in daily travel distance (from 20.0 to 8.0 km; SD 9.4). Mean positive affect similarly declined by 0.3 SD (from 3.0 to 2.79; SD 0.6), while, correspondingly, mean negative affect increased by 0.3 SD (from 1.85 to 2.10; SD 0.6). Though mobility levels partially recovered beginning in the summer of 2020, daily locations visited remained slightly below 2019 levels through the study’s conclusion in April 2022 (standardized β=–0.10; *P*<.001). Average positive affect similarly remained slightly below (standardized β=–0.20; *P*<.001) and negative affect slightly above (standardized β=0.14; *P*=.04) 2019 levels through April 2022. Weekly county-level COVID-19 transmission rates were negatively associated with mobility and positive affect and positively with negative affect, though these effects were greatly weakened later in the pandemic (eg, early 2022) or when transmission rates were high (eg, >200 new cases per 100,000 people per week).

**Conclusions:**

Findings demonstrate large initial declines in daily mobility, a moderate decline in positive affect, and a moderate increase in negative affect following the pandemic’s onset in 2020. Though most effects attenuated over time, affect and mobility levels had not recovered to prepandemic levels by April 2022. Findings support theories of hedonic adaptation and resiliency while also identifying lingering emotional and behavioral consequences. The study highlights both youth’s resiliency in adapting to major stressors while also underscoring the need for continued support for youth mental health and psychosocial functioning in the pandemic’s aftermath.

## Introduction

Individuals throughout the world faced widespread disruptions and uncertainty during the COVID-19 pandemic. From January 2020 to March 2023, the United States confirmed more than 100,000,000 cases and 1,100,000 deaths from COVID-19 [1]. Beginning in March 2020, many states closed schools and nonessential businesses and encouraged residents to minimize trips away from home [2]. Youths (ie, late adolescents and emerging adults) in particular faced unique stressors during the pandemic, most notably disruptions to developmental milestones, such as transitioning into college or the workforce, peer group formation, and parental separation [3-5], often with significant impacts on their mental health, including increases in rates of self-reported anxiety and depression symptoms [6-8].

Consistent with these mental health impacts, modest declines in positive emotional experiences and increases in negative emotional experiences following the pandemic’s onset are widely reported, though stronger impacts have been reported for adolescents and younger adults, especially in response to stressful pandemic-related experiences [9-14]. While such findings have been widely replicated, studies of the pandemic’s psychological impacts are frequently limited by measurement and study design concerns. Most lack pre–COVID-19 data, and report perceived changes in emotional well-being, which may be especially prone to recall biases in the context of traumatic events like the pandemic [15,16] rather than actual changes in emotional experiences before and after the pandemic’s onset. Furthermore, such studies were generally collected at only a few time points, often during the first few months of 2020, limiting their ability to observe changes in well-being over the multiyear course of the pandemic.

Psychological theories of adaptation to stressful experiences offer different predictions on the persistence of pandemic-related distress. Hedonic adaptation theory [17] predicts that, following stressful life events, emotional well-being may initially be substantially impacted but will recover to a pre-existing “set point” over the subsequent months or weeks [18,19]. This theory predicts that pandemic-related impacts on emotional experiences gradually attenuated over subsequent weeks and months as individuals adjusted to the event. Contrastingly, theories regarding the psychological impact of traumatic experiences assert that sufficiently distressing events may have lasting emotional consequences, suggesting many individuals may have faced lasting distress in the pandemic’s aftermath [20,21].

One response to the challenge of validly capturing pandemic-related impacts via retrospective self-report was the use of behavioral “proxy” measures, like smartphone-based GPS location data, for which prospective data were available and which did not use self-report [22]. Such data can capture features of daily routine mobility patterns, including their frequency, distance, duration, and regularity [23-26]. This research has identified widespread changes in mobility patterns following the pandemic’s onset and the implementation of lockdown policies, including fewer locations visited and shorter travel distance, and frequently a gradual recovery toward prepandemic mobility patterns following the first few months of the pandemic [27-33]. Several studies found that mobility changes were moderated by factors like local pandemic severity and sociodemographic characteristics like age, race or ethnicity, and income levels [28,29,32,34-36].

Pandemic-related mobility disruptions are widely studied among adults, though little research has investigated effects on youth mobility. Youths are less risk averse than adults [37,38], experienced lower risk of severe illness from COVID-19 [39], and were likely differentially impacted by pandemic-related policies (eg, school closures). Hence, youths and adults likely experienced a different set of salient restrictions and motivations impacting their daily routines.

GPS location data are useful for identifying changes in daily routines, though they cannot capture the pandemic’s psychological impacts. Such impacts are predominantly studied via self-report instruments. However, as previously discussed, the rapid onset of the pandemic precluded prospective data collection for many studies, leading much of the literature on COVID-19–related psychological impacts especially prone to recall biases. Ongoing longitudinal studies with prospectively measured psychological surveys, especially those conducted over multiple years before and during the pandemic, are key sources of such prospective data. Such studies are well suited to improve and expand upon the COVID-19 literature, allowing for the measurement of the pandemic’s impact on psychological outcomes with greater robustness to self-reporting biases and greater information on the persistence of pandemic-related effects.

This study investigated the magnitude and persistence of changes in youth affect and daily mobility patterns following the COVID-19 pandemic, using GPS mobility data and biweekly affect surveys collected for up to 70 months between June 2016 and April 2022 from a sample of Colorado-based adolescent and young adult twins. These data are unique in that they pair multiple years of continuously collected intensive longitudinal behavioral surveys and persistent GPS location data, with data collection beginning years before and concluding years after the COVID-19 pandemic’s onset, allowing for fine-grained analyses of changes in mood and daily routine both prior to and during the pandemic’s multiyear course. Investigating initial pandemic-related impacts on youth affect and mobility and the persistence of these changes over subsequent years, this study aimed to inform ongoing efforts to both understand and mitigate the lasting consequences of pandemic-related experiences on youth mental health and psychosocial functioning.

We had 2 primary aims. First, to investigate the magnitude of COVID-19–related disruptions to youth daily routines, measured via GPS-based mobility measures, and emotional experiences, measured by biweekly surveys measuring positive and negative affect, and the persistence of these effects over the pandemic’s multiyear course. Second, we estimated the effect of local pandemic severity (measured by the weekly incidence of county-level COVID-19 cases) on daily routine and affect as well as whether case count effects differed over the course of the pandemic.

Consistent with similar work in adults [32], we predicted sharp declines in measures of daily mobility in March 2020, following the implementation of COVID-19 mitigation policies in many US states, and a gradual return to prepandemic mobility patterns over the following months, as COVID-19–related restrictions eased, vaccines became available, and attitudes toward social gatherings grew more permissive. Similarly, we predicted moderate to large declines in positive affect and increases in negative affect in early 2020, with a return to baseline over the following months due to hedonic adaptation and increasing opportunities for enjoyment and social engagement with the loosening of pandemic-related restrictions. Finally, we predicted reduced mobility and poorer emotional well-being in areas with greater COVID-19 transmission rates and that this relationship would be strongest during the initial months of the pandemic, reflecting greater fear and uncertainty about the pandemic’s impacts.

## Methods

### Participants

Potential CoTwins participants were identified from birth records maintained by the Colorado Department of Health and Human Services as twins between the ages of 14 and 17 years upon recruitment. Recruitment procedures included both English and Spanish language digital and physical advertising as well as phone-based recruitment by study research assistants. Participants in this study were 887 twins who, at the time of their initial intake visit, were between the ages of 14 and 17 years, enrolled in a Colorado high school, and owned their own Android or iOS smartphone device (see Table 1 for participant demographics).

Parents and children both provided informed consent and assent prior to participation, and the study was approved by institutional review boards at both the University of Minnesota and the University of Colorado, Boulder. Intake visits for the first 670 participants were conducted between April 2015 and October 2016 on the University of Colorado, Boulder campus. These participants were initially recruited to participate for 1 year with the opportunity to continue for an additional year. Participating youths and their parents provided demographic information and baseline youth or parent-report data during an initial in-person assessment visit, which included cognitive testing, interviews, and both youth and parent-report questionnaires [40]. Youth participants then completed routine surveys and provided GPS data via a smartphone app for the duration of their participation in the study. A second wave of recruitment occurred between October 2018 and July 2021, with participants agreeing to participate for an additional 3 years. Of the original 670 twins, 76.1% (n=510) participated in this wave, and 217 new participants were recruited. Due to COVID-19 university-mandated lockdowns, no intake visits occurred between February 2020 and February 2021, though new participants were still recruited at this time, completing both baseline and follow-up surveys remotely via the CoTwins smartphone app (Vrieze lab). Data collection was completed in April 2022.

**Table 1:** CoTwins sample demographics (N=887)^a^.

		Values
**Sex, n (%)**		
	Female	478 (53.9)
	Male	409 (46.1)
**Race, n (%)**		
	American Indian or Alaska Native	12 (1.4)
	Asian	4 (0.5)
	Black or African American	12 (1.4)
	Native Hawaiian or other Pacific Islander	2 (0.2)
	White	715 (80.6)
	More than 1 race	90 (10.2)
	Declined to provide	56 (6.3)
**Ethnicity, n (%)**		
	Hispanic or Latino	138 (15.6)
	Not Hispanic or Latino	749 (84.4)
**Annual family income (US $), n (%)**		
	Less than $30,000	30 (3.4)
	$31,000-$60,000	98 (11.0)
	$61,000-$100,000	162 (18.3)
	$100,000-$150,000	208 (23.4)
	Greater than $150,000	225 (25.4)
	Declined to provide	164 (18.5)
**Highest attained parent education, n (%)**		
	Less than high school	4 (0.1)
	High school diploma or graduate equivalency degree	42 (4.7)
	Some college or associate degree	230 (25.9)
	Bachelor degree	310 (34.9)
	Master degree or higher	249 (28.1)
	Declined to provide	52 (5.9)
**Age (years), mean (SD)**		
	Age on January 1, 2020	19.18 (1.54)

^a^Participants were 887 adolescent and young adult twins initially recruited as high school students in the state of Colorado who provided affect surveys and persistent GPS location data over multiple years between June 2016 and April 2022. Participant demographic information was collected via parent report during an initial in-person intake assessment.

### Procedure

The CoTwins study’s primary aim was to investigate the development of substance use and executive functions in adolescence and emerging adulthood. The study used a twin design to allow genetic and environmental influences on these behaviors to be quantified. Upon enrolling in the study, CoTwins participants completed an in-person intake visit on the University of Colorado, Boulder campus that included baseline assessments and the installation of the CoTwins smartphone app. This app was then used to administer regular self-report assessments and collect time-stamped GPS location data for the duration of the study. Study staff regularly reviewed questionnaire completion rates and GPS data collection and contacted twins to assist with technical issues as needed. Additional information on the CoTwins study procedure is available in both Alexander et al [23] and Freis et al [40].

### Measures

#### Positive and Negative Affect

Positive and negative affect surveys were deployed to participants’ smartphones once every 2 weeks via an abbreviated form of the Positive and Negative Affect Schedule (PANAS) [41,42]. The abbreviated PANAS consisted of 5 items assessing negative affect and 5 items assessing positive affect. Example items include “indicate to what extent you have felt afraid over the past few days” (negative affect) and “indicate to what extent you have felt inspired over the past few days” (positive affect). Each PANAS item was answered on a 5-point scale from 1=very slightly to 5=extremely. The average past week positive and negative affect were both computed by taking the mean score of all the positive or negative items.

#### Daily Mobility Measures

Participant location was collected via the CoTwins smartphone app, installed on each twin’s personal phone. To limit battery drain, the “significant change” location application programming interface was used on iOS devices, such that a participant location was recorded each time the device registered a “significant” change in location (eg, a movement greater than roughly 100-200 m). On Android devices, the app was designed to record the participant’s location once every 5 minutes.

After visual inspection and data cleaning steps, which included removing duplicated, incomplete, inaccurately measured, or highly improbable locations (such as those implying participant movement speeds greater than 600 km per hour), locations were aggregated into points of interest, also called “staypoints,” which were locations where participants were estimated to have spent at least 30 minutes within a 200 m radius [43]. This was done to help standardize the number and meaning of GPS locations observed each day between Android and iOS participants or between participants living in urban or rural areas. All locations recorded outside the United States were removed from analyses. This allowed for harmonization with COVID-19 case count data, ensured consistent data quality and availability, and reduced possible confounding effects due to heterogenous policies, cultural environments, and atypical travel experiences. From an initial sample of 42.0 million unique GPS locations, data cleaning and aggregation procedures yielded a dataset of 2.1 million staypoints from 598,966 participant days.

Two daily mobility measures were computed from these staypoints: daily locations visited, defined as the number of staypoints recorded by a participant on a given day, and daily travel distance, the straight-line distance (in kilometers) between a day’s consecutively recorded staypoints. Travel days, in which the daily travel distance exceeded 500 km, were excluded from analyses to reduce the effect of outlying values. Additional information on the computation and measurement properties of these measures is available in Alexander et al [23].

#### COVID-19 Case Count

The Johns Hopkins Coronavirus Research Center provided daily data on the number of new COVID-19 cases recorded in each US county between January 20, 2020, and March 10, 2023 [1]. These were mapped to participants’ modal county each day (defined as the county where a participant recorded the greatest number of staypoints) to measure the degree of COVID-19 transmission in a participant’s environs. To help account for reporting artifacts (eg, counties reporting weekend cases on the following Monday), daily county-level case count was aggregated by week and standardized by the county’s population as weekly county-level case count per 100,000 people.

#### Date

A date term was used to model changes in affect and mobility over time both before and during the pandemic. This was included in statistical models as the number of days since January 20, 2020, the date of the first US COVID-19 case. Data collected prior to pandemic onset were assigned negative date values. Several follow-up analyses included a categorical effect of date to compare affect and mobility levels between January and April (corresponding to the first 4 months of the COVID-19 pandemic) in 2019-2022. Categorical date effects were restricted to data from January to April each year. This was done to account for seasonality effects, to isolate the especially large effect of the first months of the pandemic in early 2020, and to account for the lack of available data after April 18, 2022. This categorical date variable had 4 levels: indicating whether an observation was recorded from January 20 to April 30, 2019, from January 20 to April 30, 2020, from January 20 to April 30, 2021, or from January 20 to April 18, 2022 (the final day with available affect or mobility data in the study).

#### Model Covariates

Participant sex was coded as male (0) or female (1). Participant age on January 20, 2020 (included as a constant to reduce collinearity with date effects) was recorded in years. Weekday or weekend status was coded as weekday (0) or weekend (1). The operating system was recorded as either Apple iOS (0) or Android OS (1).

#### Missing Data

To help account for any systematic missingness in either the affect or mobility measures, a common challenge in intensive longitudinal research [44], we included the proportion of missing days of location data and the proportion of missed PANAS responses as model covariates. The proportion of missing days of location data was defined as the proportion of days between a participant’s first and last recorded location with no recorded staypoints. The proportion of missed PANAS surveys was computed as 1 minus the number of recorded affect surveys divided by a “theoretical maximum” number of affect surveys a person could have recorded, defined as the number of surveys a participant would have completed if they had completed 1 affect survey every 2 weeks between the date of their first and last survey response (rounded upward to the nearest integer to prevent fractional values).

### Ethical Considerations

All study protocols were reviewed and approved by institutional review boards at both the University of Minnesota (STUDY00000748) and the University of Colorado, Boulder (14-0433), to ensure compliance with ethical standards for the treatment of research participants. Participants’ parents provided written consent, and participants provided verbal assent during the study recruitment and prior to their initial baseline visit. Data were securely stored on both local encrypted devices at the Universities of Minnesota and Colorado, Boulder, and on the remote encrypted data storage platform, Box. Data identifiers, including participants’ names, birthdates, and home addresses, were stored separately from their behavioral and GPS data, which were assigned anonymized individual and family ID codes (to retain twin pair relationships in the data). Raw GPS data, which potentially allow for the inference of participant addresses and other significant locations, were deemed inherently identifying and were thus subject to additional privacy protections, including a separate participant ID, which could only be reconciled with participants’ behavioral data using a securely stored ID mapping available only to the study investigators. Only location metadata, which were not inherently identifying, including the number of daily locations visited and daily travel distance, were considered in this investigation or paired with participants’ behavioral data. Participants were compensated US $100 for their first baseline visit (plus reimbursement for any travel costs), up to US $150 (for completion of all surveys and complete GPS location data) per year of participation, and an additional US $100 after offboarding from the study.

### Analyses

All models reported in the Results section are presented in Table 2 along with their corresponding interpretation. A variety of statistical approaches were considered to model changes in affect and mobility patterns following the onset of the COVID-19 pandemic and in response to local COVID-19 cases, including linear mixed effects models, structural equation–based latent growth curve models, and generalized additive mixed models (“GAMMs”). Mobility and affect data were correlated at the individual and family level, were collected frequently at unevenly spaced intervals, and were expected to exhibit highly nonlinear trajectories both over time and in response to local COVID-19 cases. We therefore determined that GAMMs were best suited to accommodate these correlated, time unstructured data and expected nonlinear relationships [45,46].

GAMMs were fit using the *gamm4* package in R (R Foundation for Statistical Computing) [46]. Models are presented in Table 2. Linear fixed effects included sex, age, smartphone operating system, and the proportion of missing location and survey data. Random intercepts of individuals nested within families and nonlinear “smooth” terms (fit via penalized regression splines) of either date or case count were also included. Models of daily mobility further included a covariate, indicating whether the observation was recorded on a weekday or a weekend. Fixed effects covariates of both month and season were considered to correct for possible seasonality in affect and mobility patterns, though these were ultimately excluded due to a lack of sufficient prepandemic affect data to infer typical seasonal trends (particularly for affect data, which were first available in February 2019), as well as collinearity between season, COVID-19 onset, and COVID-19 case counts. Smooth effects for date were fit using thin plate regression splines with *k*=35 basis functions, while smooth effects for case counts were fit with *k*=10 basis functions. Selection of the number of basis functions was supported via the *k*-basis dimension test to prevent model underidentification [46,47]. To assess whether mobility and affect were significantly increasing or decreasing at a given date or case count level, we computed first derivatives and 99% CIs of the smooth date and case count terms at 10,000 equally spaced points on the smooth’s curve using the R package *gratia* [48,49].

Such GAMMs are useful for visualizing complex mean trajectories, but they consequently do not provide readily interpretable fixed effects coefficients for date or case count effects. Hence, complementary to these generalized additive models, we further fit linear mixed effects models, via the R package *lme4* [50], which included all fixed effects covariates included in the GAMMs, nested random intercepts for individuals nested within families, and both fixed and random effects of the year (see the Measures section) or local COVID-19 transmission rates. Similarly, to assess whether affect or mobility differed in their responsiveness to the local case count over time, we also fit mixed effects models with these same covariates along with a linear case count effect, year effect (where year indicated whether an observation was recorded in 2020, 2021, or 2022), and a case count×year interaction effect (see Table 2 for formal and text-based descriptions of each model). In an attempt to control for seasonal effects, only data collected from January through April in 2019-2022 (coinciding with the largest pandemic-related impacts in 2020) were included in these linear mixed effects models.

We considered several different strategies to reduce the impact of missing surveys and location data. The proportion of missing affect surveys and missing days of GPS location data for each participant were recorded as model covariates. Relationships between missingness, participant demographics, and affect and mobility measures were explored to identify possible attrition effects on outcomes. Additional strategies were considered, such as running analyses in a latent growth curve modeling framework with full information maximum likelihood estimation [51] or multiple imputation of missing data. However, given that predictor variables were only rarely missing, measurement occasions were irregularly spaced, and affect and mobility trajectories were highly nonlinear, we concluded that GAMMs remained better suited to modeling these relationships [45,46]. Similarly, multiple imputation was ultimately not used due to computational constraints and to the large number of missing days of data, implying a large fraction of missing information impacting the accuracy of imputation results [52]. Finally, additional biometric “ACE” decomposition models [53] were devised to investigate the extent to which COVID-19 experiences and corresponding affect and mobility changes were subject to genetic influences, though these models were ultimately not used after initial efforts demonstrated inadequate statistical power (eg, heritability estimates for local COVID-19 case counts that ranged from 0% to 100%) when estimating monthly and quarterly biometric variance parameters (identifying changes in genetic and environmental contributions over time, a significant difference between 2 or more variances, was further underpowered and typically requires thousands of twin pairs, rather than the hundreds available in CoTwins).

**Table 2:** Description of models, terms included, and related research aims^a^.

Research aim	Model	Terms
Quantify mean changes in affect or mobility over time or across COVID-19 transmission levels.	Y_*ij*_=*f*_1_(u_1__*ij*_)+X_1__*ij*_β_1_+Z_1__*ij*_b_1_+ε_*ij*_ (1)	Y_*ij*_: Vector of participant *i* in family *j*’s affect or mobility *f*_1_(u_1*ij*_): Smoothing function representing the nonlinear effect of date or local case counts on affect or mobility X_1*ij*_β_1_: Linear effect β_1_ of covariates X_1*ij*_ Z_1*ij*_b_1_: Random effects (intercept) b_1_ for individual *j* nested in family *j* ε_*ij*_: Random error term
Obtain parameter estimates for affect and mobility changes by year and across COVID-19 transmission levels.	Y_*ij*_=X_2__*ij*_β_2_+Z_2__*ij*_b_2_+ε_*ij*_ (2)	Y_*ij*_: Vector of participant *i* in family *j*’s affect or mobility X_2*ij*_β_2_: Linear effect β_2_ of covariates X_2*ij*_, including an effect of year or local case count Z_2*ij*_b_2_: Nested random effects b_2_ of individual i within family *j*, including a random slope of year or local case count ε_*ij*_: Random error term
Test whether the effect of local COVID-19 case counts on affect or mobility is moderated by year.	Y_*ij*_=X_3__*ij*_β_3_+Z_3__*ij*_b_3_+ε_*ij*_ (3)	Y_*ij*_: Vector of participant *i* in family *j*’s affect or mobility X_3*ij*_β_3_: Linear effect β_3_ of covariates X_3*ij*_, including an effect of year, local case count, and a year by local case count interaction term Z_3*ij*_b_3_: Nested random effects b_3_ of individual *i* within family *j*, including random slopes of year and case count ε_*ij*_: Random error term

^a^Description of each specific research aim, the corresponding statistical model, and definitions for each term within each model. Models were run using data provided by 887 adolescent and young adult twins initially recruited as high school students in the state of Colorado, who provided biweekly affect surveys and persistent GPS location data over multiple years via smartphone between June 2016 and April 2022.

## Results

### Descriptive Statistics

Descriptive statistics for affect and mobility measures are provided in Table 3. To assess the extent of reliability of the remote affect surveys over the multiyear duration of the study, Cronbach αs were computed at each assessment for participants’ first 70 remote affect surveys. These αs ranged from 0.67 to 0.91 for positive affect (mean α=0.78) and from 0.75 to 0.89 (mean α=0.83) for negative affect, indicating that affect surveys were adequately reliable for the duration of the study. Intraclass correlation coefficients (single random raters) for the affect surveys over time were 0.52 for average positive affect and 0.55 for average negative affect, indicating that individuals’ self-reported positive and negative affect varied between assessments but was moderately highly correlated with affect measurements at other time points. Mobility measure intraclass correlation coefficients were 0.14 for daily locations visited and 0.04 for daily travel distance, suggesting highly variable mobility patterns over the multiyear duration of the study. A greater percentage of both location data and affect surveys were missing for younger participants (β=–0.40 to –1.12; all *P*<.001) and Android users (β=0.97-44.05; all *P*<.001). Missing location data were significantly more prevalent in female participants (β=0.22; *P*<.001), while missing affect surveys were more common among participants (β=2.88; *P*<.001).

**Table 3:** Descriptive statistics for affect and mobility measures^a^.

Variable	Grand mean^b^		Within-subject mean^c^		N_Total_^d^	N_Participant_^e^		% Missing^f^	
	Mean (SD)	Median (IQR)	Mean (SD)	Median (IQR)		Mean (SD)	Median (IQR)	Mean (SD)	Median (IQR)
Daily locations visited	3.5 (2.1)	3.0 (2.0-5.0)	3.4 (0.9)	3.4 (2.8-3.9)	592,834	689.3 (498.4)	575.0 (271.5-1090.5)	46.1 (26.4)	40.0 (23.7-62.0)
Daily travel distance (km)	20.3 (42.0)	6.4 (0.6-21.2)	18.1 (9.4)	16.9 (11.5-23.4)	592,834	689.3 (498.4)	575.0 (271.5-1090.5)	46.1 (26.4)	40.0 (23.7-62.0)
Positive affect (0-5)	2.9 (0.7)	2.8 (2.4-3.4)	2.9 (0.6)	2.9 (2.5-3.2)	15,501	23.3 (18.0)	19.0 (8-36)	47.1 (24.7)	50.0 (25.0-66.8)
Negative affect (0-5)	2.0 (0.8)	1.8 (1.4-2.6)	2.1 (0.6)	2.0 (1.6-2.5)	15,500	23.3 (18.0)	19.0 (8-36)	47.1 (24.7)	50.0 (25.0-66.8)

^a^Descriptive statistics characterize data provided by 887 adolescent and young adult twins initially recruited as high school students in the state of Colorado, who provided biweekly affect surveys and persistent GPS location data via smartphone over multiple years between June 2016 and April 2022.

^b^The average value for a measure across all participants and days.

^c^The mean of participant-mean values for a measure.

^d^The total number of recorded observations across all participants for a measure.

^e^The average number of observations recorded per participant.

^f^The mean participant’s portion of missing days of location data or completed affect surveys (see the Missing Data section for additional information on missingness).

### Change Over Time and Pandemic Onset Effects on Affect and Mobility

Table S1 in Multimedia Appendix 1 reports the results of GAMMs of daily locations visited, daily travel distance, positive affect, and negative affect conditioned on a smooth date term, fixed effects covariates, and random intercepts of individuals nested in families. Significant fixed effects of covariates are reported in Table S1 in Multimedia Appendix 1 and included male sex (positive affect and negative affect), age on January 20, 2020 (locations visited and travel distance), weekend day (locations visited and travel distance), Android OS (locations visited, positive affect, and negative affect), percent missing location data (locations visited and travel distance), and percent missing affect surveys (locations visited and travel distance). Effective degrees of freedom tests of nonlinearity [47] indicated that date smooths were significantly nonlinear for all outcome variables (*F*_31.2,33.8_=29.0-400.7; all *P*<.001), while *k*-basis dimension tests [47] suggested that basis dimensions were sufficient to prevent underfitting (*k*=0.98-1.01; *P*=.14-.81).

**Figure 1:**
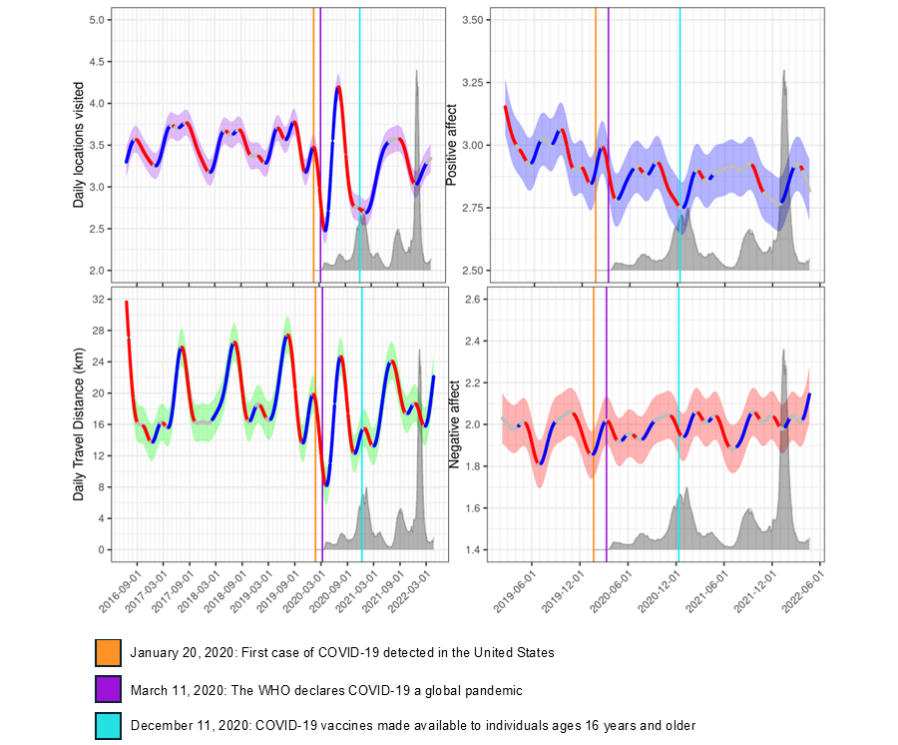
Pandemic onset effects on affect and mobility measures over time. Affect and mobility measures regressed onto a smooth date term (fit via penalized spline regression). Participants were 887 adolescent and young adult twins initially recruited as high school students in the state of Colorado who provided biweekly affect surveys and persistent GPS location data over multiple years via smartphone between June 2016 and April 2022. Colored bands indicated 95% CIs. Blue segments of the curve indicate areas where the derivative of the curve is significantly greater than 0 (*P*<.01), while red segments indicate areas where the derivative is significantly less than 0 (*P*<.01). For reference, the number of daily national COVID-19 cases is presented in dark gray behind each curve. WHO: World Health Organization.

Smooth date effects (adjusted for fixed effects covariates), characterizing the change in mean affect and mobility levels over time, are presented in Figure 1. Prior to the onset of the pandemic in January 2020, mobility measures were largely stable over time, though with seasonal fluctuations of higher mobility in the summer months and lower mobility during the winter. During the first several months of the COVID-19 pandemic, from January to May 2020, both daily locations visited and daily travel distance fell sharply to their lowest levels observed during the study, falling by 29%, from 3.5 to 2.5 locations per day, and by 60%, from 20 to 8 km per day, respectively. Both mobility measures sharply rebounded during the summer of 2020, with daily locations visited increasing by 68% and daily travel distance increasing by 200%. They then plummeted between September and December, with mean daily locations visited falling from a high of 4.2 locations per day to a low of 2.8 locations per day and mean daily travel distance falling from a high of 24 km per day to a low of 12 km per day, coincident with a large outbreak of COVID-19 cases. Beginning in December 2020 and January 2021, roughly coincident with when COVID-19 vaccines were first made broadly available to adults older than 16 years of age, daily mobility measures steadily increased for much of 2021 and 2022, though with significant decreases of ~0.6 locations per day and ~8 km per day between September 2021 and January 2022, possibly reflecting the return of seasonal declines in mobility during the winter observed prior to the pandemic’s onset. By mid-April 2022, participants visited an average of 3.3 locations per day with a daily travel distance of 28 km, both of which were similar to levels observed in the spring of 2019.

During 2019, positive affect significantly increased during the summer months and significantly decreased during the fall and winter months (all *P*<.01). In contrast, negative affect significantly decreased during the summer of 2019, significantly increased in the late summer and fall, and significantly declined in the late fall and winter (all *P*<.01). Average positive affect saw modest changes following the pandemic’s onset in early 2020, initially increasing from a mean of 2.85 in January to 3.0 in February 2020 before declining to 2.79 by April 2020 (a decline of 0.3 SDs), its lowest observed level during the study period. Contrastingly, mean negative affect initially increased from 1.85 to 2.10 between January and March 2020 (an increase of 0.3 SDs) before declining slightly to 1.91 in April and May 2020, concurrent with the implementation of COVID-19 lockdown policies. Over the subsequent 2 years, positive affect remained consistently below prepandemic levels, though with significant fluctuations: declining significantly during 2 large COVID-19 outbreaks in the fall and winter of 2020 and 2021 and increasing significantly in late 2020 or early 2021 (coincident with the rollout of the COVID-19 vaccine) and in early 2022. In contrast, mean negative affect levels continued to modestly fluctuate over the subsequent months and years of the pandemic, though generally remaining slightly above levels observed prior to the pandemic’s onset.

**Figure 2:**
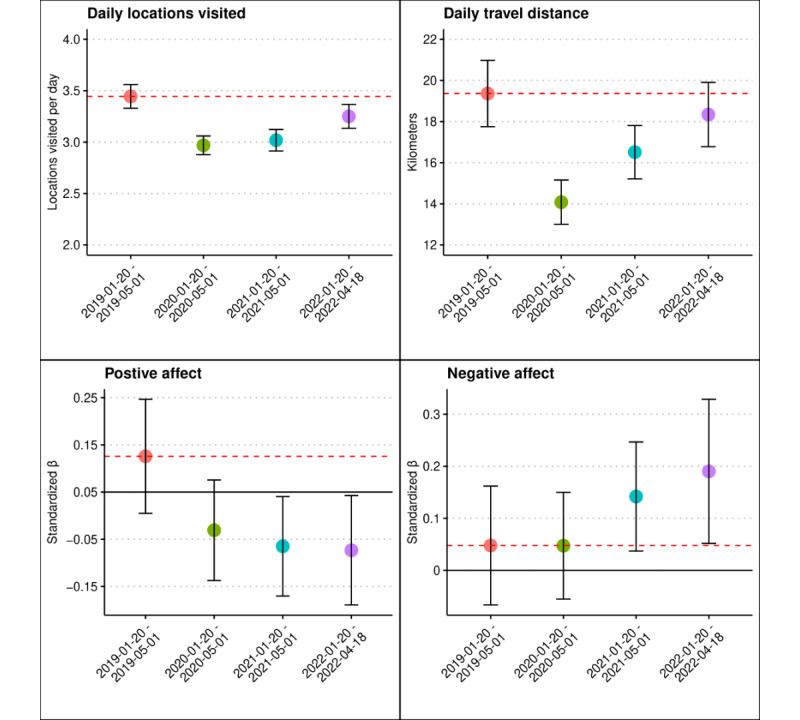
Differences in average affect and mobility: January-April 2019-2022. Predicted affect and mobility values from January 20 to May 1, 2019-2022, according to linear mixed effects models. Estimates are derived from 887 adolescent and young adult twins initially recruited as high school students in the state of Colorado who provided affect surveys and persistent GPS location data via smartphone over multiple years between June 2016 and April 2022. Affect values are represented in SD units for interpretability. Red dashed lines denote the estimated level of each outcome in 2019. Error bars represent 95% CIs.

The results of mixed effects models quantifying differences in average affect or mobility from January to April in 2019-2022 are presented in Table S2 in Multimedia Appendix 1 as well as in Figure 2. Fixed effects covariate estimates were largely consistent with those obtained via GAMMs (Table S1 in Multimedia Appendix 1). Daily locations visited between January and April 2020, 2021, and 2022 were all significantly below mean levels observed between January and April 2019 (standardized β=–0.24 to –0.10; all *P*<.001). Similarly, mean daily distance traveled remained below January-April 2019 levels in January-April 2020 and 2021 (standardized β=–0.13 to 0.07; all *P*<.001), though they were not significantly different from 2019 levels in January-April 2022 (standardized β=–0.03; *P*=.29). Mean positive affect remained significantly below early 2019 levels in January-April 2020, 2021, and 2022 (standardized β=–0.20 to –0.16; all *P*<.001). Relative to early 2019 levels, the mean negative affect was not significantly different in January-April 2020 or 2021 (standardized β=0.00-0.09; *P*=.99-.07) but was significantly higher in January-April 2022 (standardized β=0.14; *P*=.04).

### Local Case Count Effects on Affect and Mobility

Results of GAMMs of affect and mobility measures regressed on a smooth effect of local past-week COVID-19 cases per 100,000, fixed effects covariates, and random intercepts of individuals nested in families are presented in Table S3 in Multimedia Appendix 1. Fixed effects covariates showed largely the same relationships to affect and mobility measures as in the smooth date GAMMs (Table S1 in Multimedia Appendix 1). Case count smooths were significantly nonlinear for all outcome variables (*F*_5.77,8.55_=23.04-338.9; *P*s<.001). *k*-Basis tests were significant for negative affect (*k*-index=0.93; *P*<.001) and daily travel distance (*k*-index=0.97; *P*=.03), suggesting possible underfitting, though these models’ effective degrees of freedom, 6.54 and 5.77, respectively, were deemed sufficiently different from *k*, 9.00, that it was not necessary to refit these models with additional basis dimensions [47].

**Figure 3:**
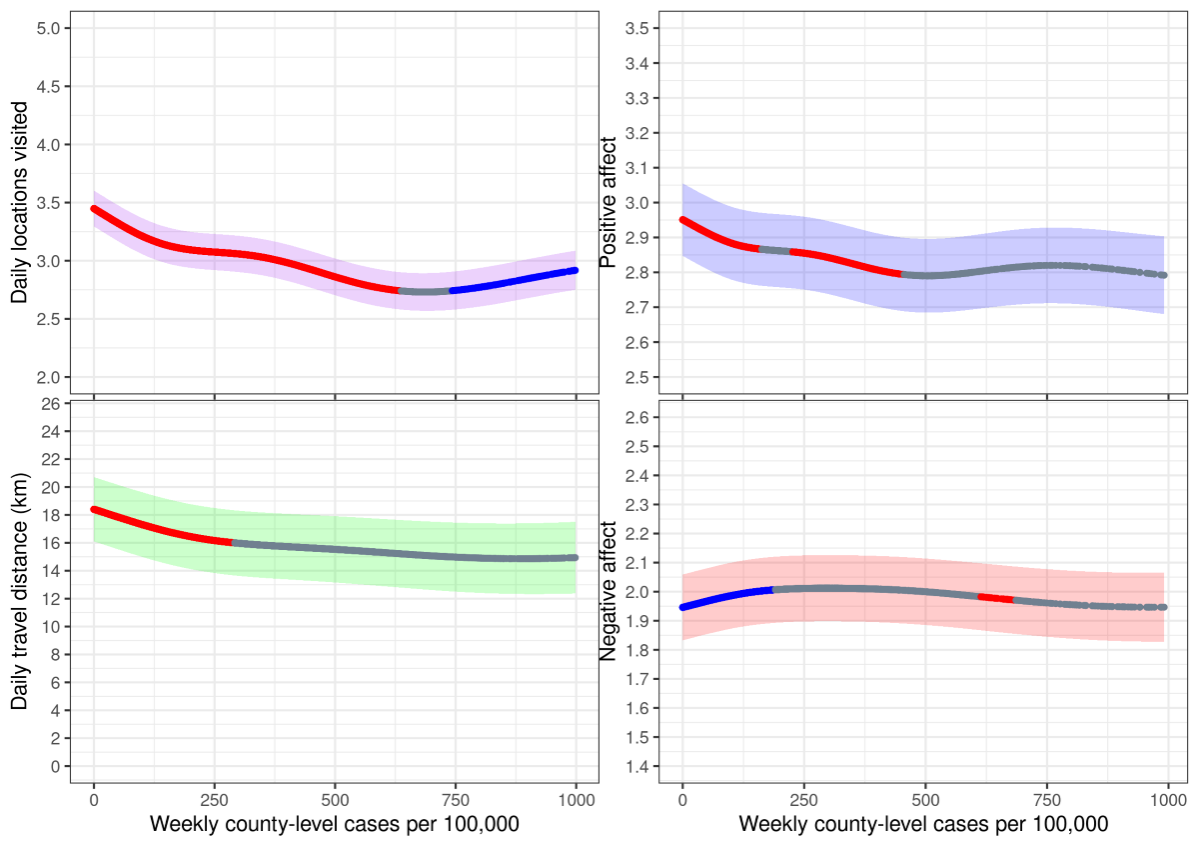
Local case count effects on affect and mobility measures. Affect and mobility measures regressed onto smooth past-week county-level COVID-19 cases per 100,000 people. Trajectories were estimated from biweekly affect surveys and persistent GPS location data provided by 887 adolescent and young adult twins initially recruited as high school students in the state of Colorado, who provided data via smartphone over multiple years between June 2016 and April 2022. Colored bands indicated 95% CIs. Blue segments of the curve indicate areas where the derivative of the curve is significantly greater than 0 (*P*<.01), while red segments indicate areas where the derivative is significantly less than 0 (*P*<.01).

The effects of local case count smooths, corrected for fixed effects covariates, on affect and mobility measures are presented in Figure 3. Increased county-level COVID-19 transmission was associated with small or moderate declines in positive affect and both measures of mobility, though increases above several hundred past-week county-level cases per 100,000 were generally not significantly related to either positive affect or mobility, with the exception of a modest but significant increase in daily locations visited at very high levels of transmission above 750 cases per participant. Correspondingly, negative affect exhibited a small but statistically significant increase of 0.06 SDs, as local COVID-19 incidence increased from 0 to 190 past-week local cases per 100,000, though further increases in local COVID-19 transmission generally exhibited nonsignificant relationships with negative affect.

**Figure 4:**
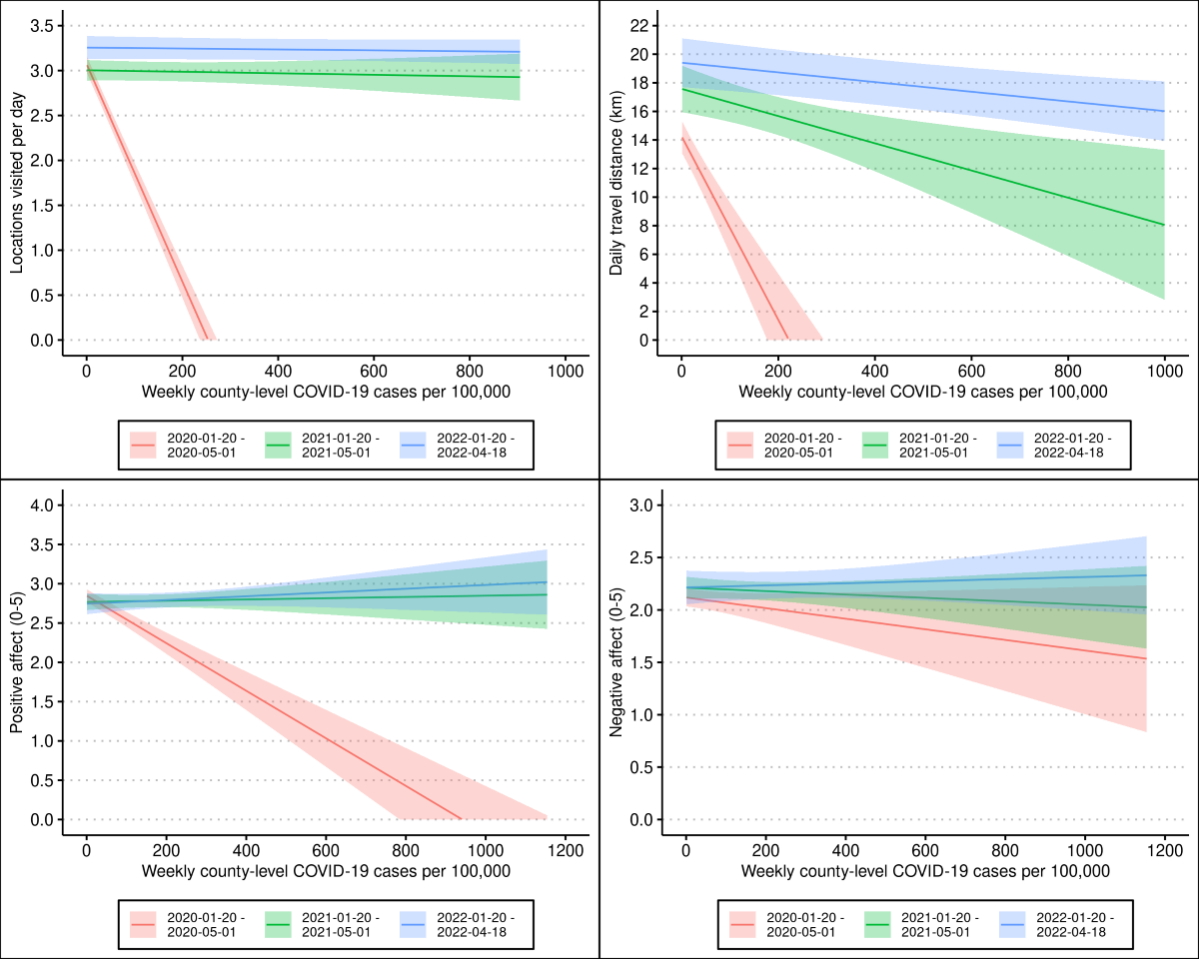
Effects of local case count on affect and mobility measures moderated by date. The linear effect of COVID-19 case counts on affect and mobility between January 20 and May 01 in 2020 (the initial phase of the COVID-19 pandemic), 2021, and 2022 obtained via linear mixed effects models. Estimates are derived from linear mixed effects models of biweekly affect surveys and persistent GPS location data collected from 887 adolescent and young adult twins, who provided data over multiple years via smartphone between June 2016 and April 2022. Shaded regions indicate 95% CIs.

Mixed effects models testing for interactions between local case count and the year in which the observation was reported are presented in Table S4 in Multimedia Appendix 1 and visualized in Figure 4. Covariate effects were largely consistent with those observed in previous models. An increase of 100 additional weekly cases per 100,000 was associated with significantly greater reductions in daily locations visited, daily travel distance, and positive affect during 2020 than during 2021 or 2022 (standardized β=0.096-1.527; all *P*<.001) and with significantly attenuated reductions in negative affect during 2022 than during 2020 (standardized β=0.184; *P*<.001).

## Discussion

### Principal Findings and Comparison to Prior Work

This study investigated changes in affect and mobility patterns in American youths throughout the COVID-19 pandemic. As a nearly 6-year intensive longitudinal design, this study represents a lengthy and longitudinally rich investigation into the pandemic’s psychological impact on youths.

Consistent with expectations, during the first pandemic months in 2020, we observed large decreases in participants’ average locations visited per day and daily travel distance, a moderate decrease in mean positive affect, and a modest increase in mean negative affect. These changes are consistent with prior research conducted in adults [10,14,29,32,34,54], suggesting that youths exhibited behavioral and emotional changes similar to adults. However, we found that mean changes in affect during the first months of the pandemic were quite small; the average positive and negative affect between January 20 and May 1, 2020, differed by less than 0.2 SDs from average levels over the same period in 2019. These youths were, on average, resilient in the face of the pandemic’s dramatic uncertainties.

Other studies have noted small or null changes in young adults’ positive and negative affect following the pandemic’s onset across varying national contexts, including in samples of college students in the northeastern United States [12], Germany [55], and China [56] and in a national web-based survey of British youths [57]. Taken together, both our results and the broader literature suggest that, across a wide range of cultural contexts, policy environments, and COVID-19–related experiences, average impacts on youth affect were likely small in magnitude even at the pandemic’s onset. However, these small average impacts do not negate the possibility that youths exposed to particularly stressful life events during the pandemic remained vulnerable, as is suggested by several papers that find youths exhibited greater emotional lability in response to pandemic-related stressors relative to middle-aged and older adults [54,58,59].

Contrary to expectation, daily locations visited and positive affect remained significantly below and negative affect significantly above early 2019 levels through at least April 2022. This incomplete return to mean prepandemic levels more than 2 years after the pandemic’s onset is more consistent with trauma-based theories on the psychology of disasters [20,21], which predict lasting emotional impacts following traumatic experiences like natural disasters. Prior research has noted postpandemic increases in youth anxiety and depression symptoms [6-8] as well as broader secular trends of increasing youth mental health problems both before and during the pandemic [60,61]. Persistent postpandemic affective changes may be a further contributor to increasing youth mental health problems. That said, effect sizes are minimal if significant, and the lasting scar of the pandemic is of uncertain significance in the lives of these youths, at least on average.

These persistent differences—years after the pandemic onset—may reflect psychological scarring or may represent the ongoing spread of COVID-19 in April 2022. Yet, we found that the relationship between affect and case count greatly attenuated with time, showing almost no relationship with affect by 2022. Another explanation is that these differences represent developmental effects associated with participants transitioning from adolescence to early adulthood. However, existing research indicates that the transition from adolescence to emerging adulthood is associated with an increase in positive affect [5] and daily mobility [23] and a decrease in negative affect [5,62], opposite of our findings. Identifying the causes of these persistent affect and mobility differences 2 years after the pandemic’s onset is likely beyond the scope of any single study; yet, speculatively, a combination of sociocultural changes, ongoing pandemic-related stressors, and lasting psychological impacts in the aftermath of the pandemic represents reasonable initial hypotheses for contributing factors.

Our hypothesis that local case count would be associated with reduced mobility and positive affect and increased negative affect was partially supported. These effects were strong earlier in the pandemic and decayed later that year and in the months surrounding the availability of vaccines. The reasons for this change could be myriad, including vaccine availability, the loosening of COVID-19 restrictions, a better understanding of the limited effects of infection on youths, or more simply changing attitudes toward the pandemic.

### Limitations

Some limitations of this study are of note. Though we find clear changes in affect and mobility upon the pandemic’s onset and extending over multiple years because of the complexity of the relationships between COVID-19–related policies, COVID-19 spread, and participant responses (as well as a lack of comprehensive data on COVID-19–related policy implementation), it was not possible in this study to disambiguate the specific features of the pandemic driving these changes, which likely included both individuals’ reactions to the spread of COVID-19 and to state and local policies enacted to reduce the pandemic’s severity (eg, school and business closures). Participants were adolescents or young adults and tended to be wealthy, educated, and White, albeit 15.6% (n=138) of Hispanic ethnicity, largely consistent with the ethnic diversity of Colorado. Generalization to other socioeconomic or ethnic contexts may not be straightforward. Indeed, generalizability may be particularly challenging for studies of pandemic-related behavior, as experiences of the pandemic varied considerably across geographic and sociodemographic contexts [31,34,35]. Twin participants are necessarily more similar to one another in their behaviors, personalities, and life circumstances than unrelated individuals. Hence, the effective sample size of the study is likely smaller than suggested by the study (N=887). While twin samples can be used to facilitate biometric analyses, such as the extent to which behavioral phenotypes and their relationships are subject to genetic influences, this sample lacked adequate statistical power to undertake these analyses in this study, at least as they pertained to the study’s objectives to examine pandemic-related changes.

Missingness remains a particularly important challenge to the collection and interpretation of intensive longitudinal data, especially over multiple years. As expected in lengthy intensive longitudinal studies, both affect surveys and mobility data were subject to substantial missing data [63,64]. Efforts to characterize and limit this impact included covarying for the proportion of missing data in all models and reporting and investigating relationships between attrition, demographics, and model outcomes and covariates. We identified several significant relationships between attrition and demographics, affect, and mobility, suggesting that, though we partially account for missingness via a fixed effects covariate, this strategy may not have fully accounted for missingness effects, especially if missingness was influenced by unanticipated and unmeasured confounds [65].

### Conclusions

In summary, we found substantial reductions in daily mobility and modest changes in positive and negative affect following the onset of the COVID-19 pandemic in early 2020, with reductions in daily locations visited and positive affect and increases in negative affect relative to early 2019 levels persisting through at least mid-2022. We further found that increases in local COVID-19 case counts were associated with reduced mobility and positive affect as well as increased negative affect, though with diminishing effects above several hundred cases per 100,000 and weaker effects in 2021 or 2022 relative to 2020. Lasting changes in affect and mobility do not appear to be well explained by the ongoing spread of COVID-19 during mid-2022 and instead may reflect numerous other factors, possibly including lasting pandemic-related harms, sociocultural changes, or disruptions to normative trajectories in social, emotional, professional, or educational development.

## Appendix

Multimedia Appendix 1 Mixed effects model summaries.

## Abbreviations

GAMM: generalized additive mixed model
PANAS: Positive and Negative Affect Schedule

## Data Availability

The datasets generated and analyzed during this study are not currently publicly available, though deidentified data (excluding GPS coordinates) are currently being uploaded to the University of Michigan’s Inter-University Consortium for Political and Social Research web-based data repository. Data are available from the corresponding author on reasonable request.
