# Supplementary material for: Investigating the Magnitude and Persistence of COVID-19–Related Impacts on Affect and GPS-Derived Daily Mobility Patterns in Adolescence and Emerging Adulthood: Insights From a Smartphone-Based Intensive Longitudinal Study of Colorado-Based Youths From June 2016 to April 2022
Source: J Med Internet Res. 2025 Mar 17;27:e64965. doi: 10.2196/64965 (PMC11959197; doi:10.2196/64965)
Supplement: Multimedia Appendix 1 [file jmir_v27i1e64965_app1.docx]

**Table S1.** Generalized additive mixed models of affect and mobility conditioned on date.


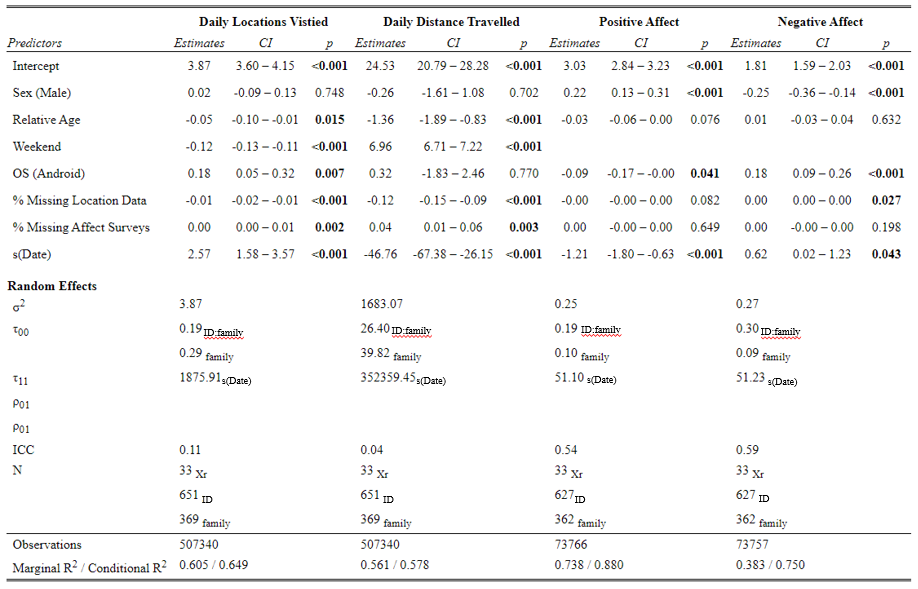


Fixed and random effects parameters from Generalized Additive Mixed models of affect and mobility conditioned on smooth effect of Date. Date was coded as the number of days before or after 01/20/2020. The smooth term was fit with k = 35 basis functions. Random intercepts were included within individuals nested in families. Bolded values indicate significant parameters at *p* < .05.

**Table S2.** Linear mixed effects models of affect and mobility from 01/20 – 05/01, 2019-2022.


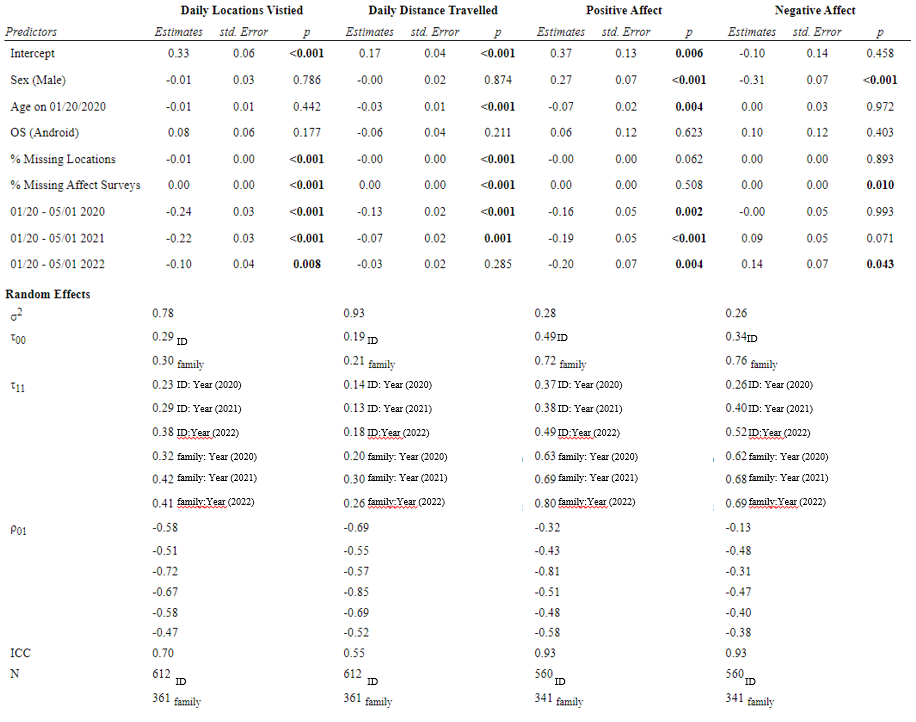


Fixed and random effects parameters from affect and mobility models investigating differences by year during the pandemic. Outcome measures were z-scored prior to analyses to allow for model convergence. Categorical year effects are relative to 01/20 – 05/01, 2019 levels. Bolded values indicate significant parameters at *p* < .05.

**Table S3.** Generalized additive mixed models of affect and mobility conditioned on local case count (past-week county-level COVID-19 cases per 100,000).


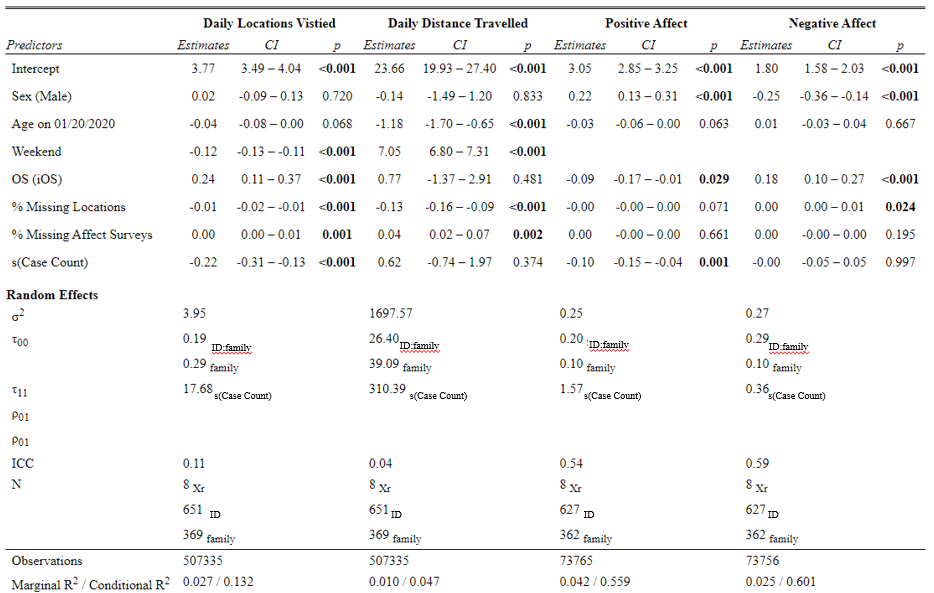


Fixed and random effects parameters from Generalized Additive Mixed models of affect and mobility conditioned on a smooth effect of local case count. Local case count was defined at the county level as the number of past week cases of COVID-19 per 100,000 residents. The smooth term was fit with k = 10 basis functions. Random intercepts were included within individuals nested in families. Bolded values indicate significant parameters at *p* < .05.

**Table S4.** Mixed effects models of affect and mobility measures: case count $\times$ year interaction models.

|  | **Daily Locations Visited** | | | | **Daily Travel Distance** | | | | | **Positive Affect** | | | | | **Negative Affect** | | | | |
| --- | --- | --- | --- | --- | --- | --- | --- | --- | --- | --- | --- | --- | --- | --- | --- | --- | --- | --- | --- |
| *Predictors* | *Estimate* | *Std. error* | | *P* | *Estimate* | | *Std. error* | | *P* | *Estimate* | | *Std. error* | | *P* | *Estimate* | | *Std. error* | | *P* |
| Intercept | -0.799 | 0.070 | | **<.001** | | -0.189 | 0.051 | | **<.001** | | -0.393 | 0.155 | | .011 | | -0.206 | | 0.153 | .176 |
| Sex (Male) | 0.007 | 0.032 | | .836 | | 0.004 | 0.021 | | .843 | | 0.246 | 0.076 | | .002 | | -0.309 | | 0.076 | **<.001** |
| Age on 01/20/2020 | -0.008 | 0.012 | | .481 | | -0.033 | 0.008 | | **<.001** | | -0.068 | 0.026 | | .009 | | 0.001 | | 0.026 | .957 |
| OS (Android) | 0.081 | 0.068 | | .239 | | -0.029 | 0.050 | | .566 | | -0.022 | 0.130 | | .869 | | 0.001 | | 0.126 | .992 |
| % Missing Locations | -0.009 | 0.001 | | **<.001** | | -0.003 | 0.001 | | **<.001** | | -0.003 | 0.002 | | .188 | | 0.000 | | 0.002 | .959 |
| % Missing Affect Surveys | 0.003 | 0.001 | | **<.001** | | 0.002 | <0.001 | | **<.001** | | 0.002 | 0.002 | | .345 | | 0.005 | | 0.002 | **.004** |
| 01/20 – 05/01, 2021 | 0.931 | 0.041 | | **<.001** | | 0.311 | 0.035 | | **<.001** | | 0.568 | 0.074 | | **<.001** | | 0.181 | | 0.067 | **.007** |
| 01/20 – 05/01, 2022 | 1.062 | 0.044 | | **<.001** | | 0.383 | 0.037 | | **<.001** | | 0.571 | 0.096 | | **<.001** | | 0.234 | | 0.097 | **.016** |
| Weekly County Covid-19 Cases per 100,000 | -1.534 | 0.052 | | **<.001** | | -0.415 | 0.053 | | **<.001** | | -1.044 | 0.108 | | **<.001** | | -0.154 | | 0.095 | .104 |
| Weekly County Covid-19 Cases per 100,000 $\boldsymbol{\times}$ 01/20 – 05/01, 2021 | 1.523 | 0.055 | | **<.001** | | 0.353 | 0.056 | | **<.001** | | 1.071 | 0.096 | | **<.001** | | 0.105 | | 0.091 | .249 |
| Weekly County Covid-19 Cases per 100,000 $\boldsymbol{\times}$ 01/20 – 05/01, 2022 | 1.527 | 0.053 | | **<.001** | | 0.393 | 0.053 | | **<.001** | | 1.126 | 0.098 | | **<.001** | | 0.184 | | 0.092 | **.045** |
| **Random Effects** | **Var_Family_** | | **Var_Indiv_** | | **Var_Family_** | | | **Var_Indiv_** | | **Var_Family_** | | | **Var_Indiv_** | | **Var_Family_** | | | **Var_Indiv_** | |
| Intercept | 0.062 | | 0.066 | | 0.017 | | | 0.014 | | 0.294 | | | 0.784 | | 0.253 | | | 0.603 | |
| 01/20 – 05/01, 2021 | 0.133 | | 0.086 | | 0.019 | | | 0.068 | | 0.178 | | | 0.657 | | 0.305 | | | 0.661 | |
| 01/20 – 05/01, 2022 | 0.093 | | 0.128 | | 0.005 | | | 0.001 | | 0.452 | | | 1.060 | | 0.372 | | | 1.051 | |
| Weekly County Covid-19 Cases per 100,000 | 0.004 | | 0.012 | | 0.059 | | | 0.004 | | 0.199 | | | 1.363 | | 0.171 | | | 0.632 | |
| **Random Effects Correlations** | **Cor_Family_** | | **Cor_Indiv_** | | **Cor_Family_** | | | **Cor_Indiv_** | | **Cor_Family_** | | | **Cor_Indiv_** | | **Cor_Family_** | | | **Cor_Indiv_** | |
| Intercept:  01/20 – 05/01, 2021 | -.577 | | -.665 | | .240 | | | -.421 | | -.580 | | | -.602 | | -.967 | | | -.501 | |
| Intercept:  01/20 – 05/01, 2022 | -.506 | | -.576 | | -.040 | | | -.354 | | -.737 | | | -.737 | | -.463 | | | -.521 | |
| Intercept:  Weekly County Covid-19 Cases per 100,000 | -.723 | | -.468 | | -.445 | | | .524 | | .320 | | | .604 | | .711 | | | .403 | |
| 01/20 – 05/01, 2021:  01/20 – 05/01, 2022 | .349 | | .518 | | .098 | | | .662 | | .624 | | | .823 | | -.501 | | | .678 | |
| 01/20 – 05/01, 2021:  Weekly County Covid-19 Cases per 100,000 | .710 | | .533 | | -.265 | | | -.399 | | -.215 | | | -.701 | | -.521 | | | -.605 | |
| 01/20 – 05/01, 2022:  Weekly County Covid-19 Cases per 100,000 | .804 | | .570 | | .851 | | | -.567 | | -.800 | | | -.656 | | .403 | | | -.593 | |

Fixed and random effects parameters from affect and mobility models investigating moderation of case count effects by year during the pandemic. Outcome measures and Covid-19 case count effects were z-scored prior to analyses to allow for model convergence. Categorical year effects are relative to 01/20 – 05/01 2020 levels.
